# Diphtheria

**Published:** 1859-11

**Authors:** W. Godfrey Dyas

**Affiliations:** Chicago


ARTICLE VI.

### DIPHTHERIA.

BY W. GODFREY DYAS, M. D., CHICAGO.

(Continued from page 605.)

The year 1855, in France, proved fruitful in cases of diphtheria. The disease then raged epidemically in that country, and it was observed that its adynamic character had become more marked. Death by asphyxiation, which, in Bretonneau's time, was the rule, now became the exception. Most of the fatal cases terminated by exhaustion. It was also observed that fever became but a secondary element in the disease—many cases proving fatal without it. As it may be instructive to give a case of this kind, we quote one detailed by M. Ramon in the *Gazette des Hopitaux*, September 25, 1855.

"A girl, aged 10 years, was brought to the Infirmerie de Charenton, presenting the following appearances: pearl-like exudations covered the posterior part of pharynx and the tonsils, which were swelled. There was no fever, nor indeed any general symptoms to be noticed. The treatment adopted seems not to have been very active, for nothing internally appears to have been administered excepting barley-water sweetened with honey, and the throat was brushed with honey of roses mixed with alum. Next day the membranous exudation was thicker, and appeared disposed to be detached from the left tonsil; the exudation was surrounded by a red border, slightly bleeding in points; the breath was not fetid, and the general state was supposed to be good. The same treatment was continued. On the fourth day of the disease, the throat was become nearly free from false membranes. In fact, the local affection presented evidently such a diminution that if it alone were to determine the prognosis, there were grounds to hope the disease would soon completely disappear. There still was no fever, yet the patient became more feeble. During the day she vomited some bile, without having previously had any disturbance of function on the part of the stomach. At the circumference of the nostril of the left side, there appeared a little whitish exudation of about a line in extent from above downwards. On the fifth day of the complaint, the tonsils and pharynx were almost entirely clean. Still no fever, but the child was growing more feeble. Deglutition painful; no appetite. Left nostril, from which there flowed some serum, was narrowed and surrounded by brownish crusts. On the sixth, the patient had a bad night, was restless and enjoyed no sleep; countenance indicated fatigue; pulse small and frequent. Some vomiting of bile. Deglutition painful; urine, thick and scanty. Tongue and throat appeared as they would in scarlatina. Some slight traces still of false membrane on left tonsil and posterior wall of pharynx. In the evening, evidently better; moderate heat of skin; pulse less frequent; tongue and fauces less red. Vomiting not renewed. There yet remained some indistinct appearance of false membrane; deglutition still painful. Chicken broth was given and not rejected by vomiting. No tympanitis, nor abdominal pain; one motion during the day. Seventh day.—Bad night, worse than had been expected; vomiting; skin cold; traits of the countenance altered; pulse small and frequent. In the evening, the left part of the velum palati and tonsil of the same side, as well as the outer wall of the left nasal fossa, were covered with a fresh membranous exudation. The breath is not fetid; skin cold; pulse always small and contracted, but less frequent than in the morning. Abdomen not tense, but is the seat of some undefined uneasiness. (Cauterization of throat with a concentrated solution of nitrate of silver). Eighth day.—Night passed without any rest; excessive debility; cold skin; intellectual faculties retain their integrity; respiration normal; death."

In the sixth volume of the *Gazette Medicale* (1838), there is to be seen a case which, in being free from fever from beginning to end, bears a marked affinity to that just now detailed. It was described as croup, and is the only one of that disease to that date on record wherein could not be detected a single febrile symptom. Cases, unaccompanied by fever, of tubular bronchitis and of pellicular disease of the mucous membrane of the intestines have long been familiar to us; but until M. Ramon placed on record his case of diphtheria without fever, we were unable to predicate universally (in reference to diseases characterized by a deposition of false membrane) that pseudo-membranous exudation, far from being necessarily accompanied by fever, may be deposited on any mucous membrane without the system giving any indication of sensorial or general vascular disturbance. M. Ramon, in laying down the four following propositions, puts on record his views of diphtheria:

1st. Diphtheria is an inflammatory disease, but the inflammation which constitutes the essence of it is allied to a peculiar condition of the system that reveals itself by a coagulation of the products of the inflamed surfaces.

2nd. The concretions that cover the inflamed surface are merely in juxta-position; they are not in union with it, and cannot therefore be confounded with gangrenous products.

3rd. Diphtheria and croup appear to be one and the same disease, the progress of which is at one time ascending—at another descending.

4th. This difference in the mode of progress of croup appears to establish one equally great in the prognosis and treatment. When the disease follows a descending progress, and this he considers as fortunately the more frequent mode, there is reason to expect it will yield to suitable external and internal remedies. If, however, it seizes on the trachea, M. Ramon thinks tracheotomy should be had recourse to. When it commences in the bronchi and ascends, he thinks then tracheotomy presents but a slender chance of relief.

These opinions, at a future stage of our observations, will engage our attention: we will not now stop to examine them, but pass on to a consideration of the disease as we find it in close relation to others.

It was during this epidemic, so rich in material for observation, that Marechal de Calvi presented his memoir to the Academie des Sciences. He looked upon the disease as having its essence in the blood; and though the exact nature of the *materies morbi* could not be ascertained, he considered the formation of false membrane as attesting an excess of plasticity of that fluid, which he endeavored to lessen by the administration of alkalies. He, therefore, gave bi-carbonate of soda in a case of this kind, and was surprised to see the effect produced in four hours—the plastic coat of the throat had completely disappeared. The quantity administered was something more than fifteen grains every half hour.

There was nothing new in this treatment. The same was proposed and tried by Mouremans in 1838. The case was diphtheria, though described as croup, and recovered.—*Medical Gazette*, April 13, 1839.

We have hitherto been considering this disease without complication. But the epidemic of 1855 did not confine itself to cases free from other complaints. It complicated scarlatina, pertussis, scurvy, dysentery and typhoid fever.

At the Hopital Saint-Antoine it was observed by M. Oulmont as a complication of typhoid fever. This physician tells us he never saw diphtheria at the above hospital seize on persons in good health or in convalescence. In all cases, it was seen complicating some serious affection, on which it supervened, and hastened its fatal termination. It affected a remarkable predilection for typhoid fever. Of eight cases of diphtheria, six appeared in patients labouring under typhoid fever, one in a man ill of double pneumonia, and one the subject of tubercular peritonitis. It appeared, moreover, at variable periods; generally between the second and fourth week of the fever; very rarely towards its decline. In most cases, it accelerated the fatal termination, which was the immediate result of asphyxia or of a kind of poisoning. In but two of these cases was there a cure. Typhoid fever, in every instance, was unfavorably influenced by the complication, and the latter developed itself without making its approach in that insidious manner to which authors have called attention. Five patients complained of a little uneasiness of the throat and difficulty of deglutition, with swelling of the cervical glands. This last symptom observed by most writers as almost always constant, was wanting in the other patients. In these it was only by an examination of the throat that the disease could be recognised. Then on the uvula, velum, posterior surface of the pharynx, might be seen irregular plates of a greyish hue, very soft, though adherent. That part of the fauces not covered with diphtheritic patches was of a bright red and swelled, as if there was submucous œdema. The tonsils were enlarged in but half the cases. In the rest they were almost normal, except for some shreds of membrane, and it was in these cases that no swelling of the cervical glands, nor of those immediately below the jaw, could be detected. Once the membrane was developed, it rapidly spread over the fauces, and in some hours the guttural fossæ were lined with it. It did not extend to the nasal fossæ. Sometimes, in about twenty-four hours it would extend to the larynx, and then repeated paroxysms of suffocation, with croupal cough indistinctly marked, would supervene. In two cases there were symptoms of extreme asphyxia. In some, the false membrane seemed to vanish from the uvula, pharynx and tonsils; there was no paroxysm of suffocation; nothing, in short, to indicate the extension of the disease to the respiratory passages; and yet, notwithstanding the apparent decline of local symptoms, there came on rapid sinking, coma and death. In two, who recovered, the false membrane in successive portions became detached, and was carried off in a thick viscid and bloody saliva, leaving the mucous membrane injected and the subjacent parts œdematous. The general symptoms of diphtheria were difficult to disentangle from those of the original complaint—so great was the confusion caused by their mutual involvement. Yet M. Oulmont referred more especially to diphtheria, the swollen face and the œdema of the side of the neck, which occasionally occurred; the tongue was generally dry and swelled; the mouth clammy, the breath fetid; diarrhœa persisted in all, even in those who recovered; the fever augmented, and the cerebral symptoms of the typhoid affection seemed to augment after the appearance of diphtheria. In all the cases, in which the disease terminated in death, its progress was very rapid. In five, it was of but two days' duration, and twice it terminated fatally by suffocation. In the others, death took place by a gradual sinking that succeeded to great excitement. In those who died, the pseudo-membrane, instead of presenting the smooth, shining and *lardacee* appearance most usually seen, was of a dirty grey, very soft and easily removed from the surface. In two of these subjects, the false membrane was limited to the uvula, tonsils and pharynx. The larynx was perfectly healthy. In two, there were but some shreds in the larynx, and in two others the membrane commencing in the back of the throat extended in a continuous line along the pharynx, larynx and trachea.

M. Oulmont adopted the treatment usually had recourse to. He used nitrate of silver both in substance and solution, and muriatic acid. But the results were not satisfactory. He thought muriatic acid caused a great accumulation of mucus above the larynx with distressing dyspnœa. Being struck with the dull-grey color of the false membrane which resembled the grey pellicle covering ulcers affected with hospital gangrene, and recollecting the satisfactory results obtained from lime juice by some surgeons in such cases, he prescribed it as a local application to the fauces in diphtheria. He directed nitrate of silver or muriatic acid to be used morning and evening, and, in the interval, lime juice. Some died so rapidly as not to give time to apply it. Thus its value could not well be appreciated. However, two, thus treated, got well: one on the twenty-sixth day of fever, the other on the fifth day of double pneumonia.

In the *Gazette Medicale* (1848), there is a case related by Barbier which bears a remarkable resemblance to those given by M. Oulmont. It is described as a case of typhoid fever complicated with laryngo-bronchial croup. It commenced like influenza with sudden seizure, coryza, etc., and terminated fatally. Though described as croup, judging from the symptoms, it was evidently a case wherein diphtheria supervened. Louis also met a case of typhoid fever similarly complicated.

Contrary to what we might suppose *a priori*, diphtheria, when complicating scarlatina, proved milder than when idiopathic. That eruptive diseases have some modifying power over the disease would seem probable from the records of cases; but that diphtheria could supplant any of the former, we had no reason to infer, knowing how frequently it was noticed in connection with scarlatina.

A paper, however, was presented to the Academy by Velpeau, in the name of M. Vernhes, physician at Beziers, entitled Simples Propositions sur le Croup, wherein under the name of croup is described this epidemic diphtheria. The author having remarked that the department of Herault, which for three years was under the influence of a "medico-croupal constitution" the most intense and fatal, during that time presented not a single case of measles. He concluded from this that these affections were to a certain point similar, and was led to think that in inducing, by artificial means, a general exanthem of the surface, it would be possible to succeed in preventing or arresting in the beginning the development of diphtheritic symptoms. He, in consequence, conceived a method of treatment, consisting in provoking a confluent eruption over the whole body by means of croton oil. He says he has proved that after the appearance of an exanthem, the formation of false membrane, if it had already taken place, was completely arrested. The fact is interesting, but the inference deduced is palpably illogical. To assume that the non-appearance of measles, under the circumstances mentioned, was owing to the presence of a disease similar in nature which, as it were, represented the former in the system, is altogether gratuitous.

While this epidemic was prevailing in France, its seeds appear to have been transmitted to the Crimea, where it broke out amongst the French troops, and appeared most frequently in complication with scurvy and diarrhœa. We are indebted to

M. Haspel, physician to the army of the East, for a most instructive account of the disease. It was particularly during the spring and autumn of 1855 that this disease appeared, complicating advanced cases of scurvy and chronic diarrhœa, especially in feeble persons, whose constitutions had been reduced either by previous or actual disease. Sometimes, localized in the superior part of the pharynx and in the tonsils, it, in the beginning, consisted frequently in but a redness more or less intense, with tumefaction of these organs, which, as well as the velum and uvula, then assumed a covering, in certain points, of pseudo-membranous white and irregular patches. Occasionally it limited itself to this, but most frequently it extended to the air passages and nasal fossæ; at other times, it presented from the commencement superficial ulcerations that were soon covered with membranous exudation. By the oppression, coughing, hoarseness, the seat of pain at the inferior part of the neck or behind the sternum, it was known that the air passages were attacked. The patients soon began to expectorate a purulent fluid, whitish and not coherent, which obstructed the bronchi and rendered respiration difficult. Two of the cases detailed, commencing as simple angina and being consecutively membranous, all at once assumed the character of gangrenous angina, in men affected with scurvy. On dissection, the disease was marked by bright-redness and swelling of the pharynx. Irregular flaps, small in size, of pseudo-membrane covered certain points of the surface of the pharynx, tonsils, velum, sometimes also the larynx and trachea. Beneath, the mucous membrane was red and rough; the epidermis had been removed, and there evidently was erosion of the surface. But on an incision being made into the trachea and bronchi, the abundance of sero-purulent liquid which had accumulated and escaped was particularly striking.

The treatment consisted in vomits. Sometimes the dysphagia was such as to cause rejection of fluids by the nares, even when taken in the smallest quantities. At other times, they acted on the intestinal tube, especially in those who had diarrhœa. Cauterization by muriatic acid, and nitrate of silver was used early in the disease but without effect. This cauterization was often difficult. It left in the throat an intolerable savour, and was not effected without danger. Besides, it produced violent excitement by the energetic efforts it provoked; and by the excessive elevation of vital manifestation it momentarily roused, exhausted the resources of the system and hastened the fatal termination, especially if, as was the case here, one had to deal with deteriorated constitutions.

Intimately connected with researches on this disease during the epidemic of 1855, a new phenomenon was made known by Bouchut and Empis. Albuminuria was observed to exist in a certain number of children affected with diphtheria. This did not appear in every case of the disease;—it was seen in eleven out of fifteen cases. It was considered to indicate the infectious nature of the disease, resembling, in that respect, purulent infection, which is accompanied by the same alteration of urine. Besides, as its disappearance coincides with the cure of the disease, it was supposed to constitute one of the most valuable signs. In the words of the report read to the Academy in reference to the memoir presented on this subject by Bouchut and Empis, albuminuria, as seen in diphtheria, "may depend on different causes, which it is necessary to seek out in order not to make any premature generalization, and to restrict us to the precise and scrupulous observation of facts. It is known that eruptions of scarlatina may precede or follow malignant angina, croup, ulcerated and gangrenous sore throat. This is the result known from cases of old, to be traced back to the time of the Syriac ulcer, so well described by Aretæus." "In this case, the albuminuria may be referred to scarlatina."

Wherever there is a stasis of the blood, as in croup with asphyxia, pertussis, cholera, affections of the heart, or any condition of the system causing congestion of the kidney, there will also be albuminuria. But in addition, there is supposed to be in diphtheria a special albuminuria depending altogether upon poisoning of the blood, one that seems to ally diphtheria to purulent infection—an albuminuria that is not connected with scarlatina, nor with asphyxia, but which belongs to the nature of the disease itself, and which announces the generalization of the latter. We, at present, content ourselves with stating the fact, but withold our assent to the opinion based on it by the authors of the paper, who pronounce it the first degree of infection of the humours by the absorption of a special purulent product which poisons the patients and causes them to die so unexpectedly.

The sudden and unexpected death may certainly be owing to the blood being saturated with a poison, but we have no evidence of a special purulent product being absorbed. The mere presence of albuminuria apart from the stasis of the blood is not the only fact that has led MM. Bouchut and Empis to the above conclusion. They assert that both in diphtheria and purulent infection there are, 1st, Alteration of the color of the blood which assumes a bistre tint; 2nd, Nuclei, more or less numerous, of pulmonary apoplexy, like those that precede the development of metastatic abscesses; 3rd, Ecchymoses of purpura on the skin, on the serous membranes and in the viscera. They think that now there is nothing more necessary to establish the connection between these diseases than the presence of visceral abscesses or purulent (metastatic) collections in the serous membranes in addition to the preceding alterations.

"But," they observe, "these abscesses are not so necessary to characterize absorption of pus, and they do not exist in all those who die of purulent infection." In all the children under their care for diphtheria, the urine was analyzed both by heat and nitric acid. When there was albuminuria, the urine contained an enormous quantity of salts, which rendered it muddy and milky-looking at the moment of emission. At first the heat caused the salts to be held in solution; then at a higher degree of heat, there was a precipitation of albumen. Three times the precipitate was remarkably abundant; once it was scarcely apparent, and in the rest of moderate amount.

The epidemic of 1855 was, as we have said, essentially adynamic in character and varied thus considerably from what Bretonneau described, which was more inflammatory and caused death by symptoms of croup, whereas that which now engages our attention destroyed patients as glanders would kill. It was contagious, developing itself slowly and insidiously, marked by prostration and debility, feeble pulse and swelled cervical glands, and existence of false membranes in the nasal fossæ, pharynx, vulva, sometimes in the larynx, trachea and bronchi. It had no gangrenous odour, was not characterized by difficult breathing, and did not destroy by asphyxia. Tracheotomy was contra-indicated. Cauterization of the surface and tonics were the only means that seemed useful. But under every plan of treatment, the majority of those ill of the disease died.

Bretonneau did not suppose that the disease he described could be propagated through the air, but supposed it was always the result of a kind of inoculation—of a real contact of the morbid products with the mucous membrane. Trousseau was less absolute in opinion. He did not reject the contagion *a distance*, which to-day is generally admitted. Bretonneau considered the descending progress as a characteristic of the disease, but this was contradicted by innumerable facts supplied by the epidemic of 1855. But though contagion could not be doubted, yet epidemic influences were the principal cause of the extension of the disease at that time.

Dr. Adolphe Gubler, as the result of extensive observation during this epidemic, was induced to view diphtheria and gangrenous sore throat as one and the same general disease, with different local manifestations; and thinks that, though one mode of expression may be the more frequent, still it is not rational to define the disease by that alone. That anatomical forms can only serve to mark peculiarities in the species; but, that in order to unite, under one denomination, these severe anginæ, whether diphtheritic, sphacelo-diphtheritic or gangrenous, we should not have regard to any character except a constant one, and designate them as malignant angina. We must here quote Guerin's comments on the above paper, they are so significant, and so much in accordance with our own views. "These reflections of M. Gubler are a still further proof of the struggle which establishes itself amongst all against the excessive predominance given among us to the anatomical element in disease. The general state of the economy claims every day its more considerable share in the physiognomy of disease. Anatomo-pathological materialism led us too far."

Before we take leave of this part of our subject, perhaps it would be well to consider more in detail the claims which the opinions and practice of Marechal de Calvi and M. Vernhes have to our confidence. We have already stated that the former, viewing the false membrane as the expression of some unknown morbid principle, and which, though itself not constituting the disease, was the immediate result of the first pathological phenomenon, and attested at least an excess of plasticity of the blood. The indication he therefore thought consisted in removing this undue plasticity, and with that view he administered alkaline carbonates; with what result we have already above stated.

Now it is a remarkable fact that during the Crimean campaign, diphtheria was seen in the French camp amongst those who at the time were suffering from scurvy. Surely it will not be said there was an excess of plasticity of their blood; nor will it be maintained there was a want of alkalies in it. We have the testimony of Fremy, Andral, Gavarret, Becquerel, Rodier and Headland, that in the blood of scorbutic patients there is an excess instead of a deficiency of the alkaline salts.

During this epidemic it was observed that diphtheria differed from other specific diseases, such as small-pox, measles and scarlatina, by the frequency of its relapses. This feature alone would discountenance the notion of similarity between it and measles, as advanced by M. Vernhes. The fact that for three years during which the "epidemic constitution" was decidedly diphtheritic in the department of the Herault, and during which there was not, in an extensive district, a single case of measles, is an important fact, and one which we would explain on an altogether different principle from that proposed. Instead of recognising any affinity between diphtheria and measles, we, on the contrary, are disposed to see nothing but opposite relations. These are frequently seen amongst diseases. Small-pox and vaccinia are a proof of the mutual counteraction of morbid processes. Ague is said to be antagonistic of phthisis; and it is alleged that Fouquet of Freiburg has successfully opposed syphilis by revaccination. M. Vernhes has given us a fact and a theory. The first is important and may lead to some practical use; the second is totally unsustained and in opposition to analogy.

During the progress of the epidemic, practitioners avoided the employment of cutaneous revulsives as they became new foci of diphtheritic deposition. There was, however, one situation, we are told, where a breach of surface was not necessarily followed by false membrane, but the contrary was observed; as we learn that benefit ensued from the excision of a tonsil. The case is related by M. Domerc. The patient was a child about five years old, and had previously been ill of whooping cough when seized with diphtheria. One of the tonsils was amputated with the effect of arresting the secretion of false membrane, and subsequently there was no deposition on the denuded surface. The treatment that was at this time usually employed in France consisted in being very sparing of sanguineous evacuations, as the affection became promptly adynamic; all cutaneous revulsives were proscribed; emetics were thought valuable; and alteratives of mercury, alkaline carbonates and chlorate of potash were supposed useful. Local treatment was directed to the fauces, with the view of energetically modifying the diseased surface—calomel, powdered alum, muriatic acid, but especially the nitrate of silver, were used. In fine, besides employing remedies to counteract the morbid principle in the blood, as each based his practice on some theory that involved this notion, restorants were freely administered both in the way of food and medicine—quina, coffee, generous wines, etc. —the employment of which had long to be continued after convalescence.

(TO BE CONTINUED).

